# Targeting Host Cellular Factors as a Strategy of Therapeutic Intervention for Herpesvirus Infections

**DOI:** 10.3389/fcimb.2021.603309

**Published:** 2021-03-19

**Authors:** Kumari Asha, Neelam Sharma-Walia

**Affiliations:** H. M. Bligh Cancer Research Laboratories, Department of Microbiology and Immunology, Chicago Medical School, Rosalind Franklin University of Medicine and Science, North Chicago, IL, United States

**Keywords:** herpesvirus, latency, lytic, arachidonic acid, antiviral

## Abstract

Herpesviruses utilize various host factors to establish latent infection, survival, and spread disease in the host. These factors include host cellular machinery, host proteins, gene expression, multiple transcription factors, cellular signal pathways, immune cell activation, transcription factors, cytokines, angiogenesis, invasion, and factors promoting metastasis. The knowledge and understanding of host genes, protein products, and biochemical pathways lead to discovering safe and effective antivirals to prevent viral reactivation and spread infection. Here, we focus on the contribution of pro-inflammatory, anti-inflammatory, and resolution lipid metabolites of the arachidonic acid (AA) pathway in the lifecycle of herpesvirus infections. We discuss how various herpesviruses utilize these lipid pathways to their advantage and how we target them to combat herpesvirus infection. We also summarize recent development in anti-herpesvirus therapeutics and new strategies proposed or under clinical trials. These anti-herpesvirus therapeutics include inhibitors blocking viral life cycle events, engineered anticancer agents, epigenome influencing factors, immunomodulators, and therapeutic compounds from natural extracts.

## Introduction

Herpesviruses are divided into three groups: The α-herpesviruses (herpes simplex virus types; HSV-1 and -2 or HHV-1 and HHV-2), and varicella-zoster virus (VZV, or HHV-3), β-herpesviruses as human cytomegalovirus (HCMV, or HHV-5), human herpesviruses 6 and 7 (HHV-6 and HHV-7), and γ-herpesviruses Epstein-Barr virus (EBV, or HHV-4) and Kaposi’s sarcoma herpesvirus (KSHV, or HHV-8). HSV-1 and VZV establish latency in sensory ganglions (Zerboni et al., 2013), and their reactivation from latency results in replication and shedding of infectious virus ready for transmission to the naive population. The α-herpesviruses HSV -1 and -2 infect a broad range of both human and non-human cells including fibroblasts, epithelial cells, and neurons (Spear and Longnecker, 2003). VZV is a both lymphotropic and neurotropic human-specific virus with highly restricted infection in other species (Zerboni et al., 2014; Oliver et al., 2016). VZV infects human neurons, skin cells, T cells, glial cells, and infiltrating macrophages (Zerboni et al., 2014; Oliver et al., 2016). β- (HCMV, HHV-6A, HHV-6B, and HHV-7) and γ- (EBV and KSHV) herpesviruses establish latent infection in cells of the immune system, differentiated lymphoid and myeloid immune cells, epithelial, and fibroblast cell (Cruz-Muñoz and Fuentes-Pananá, 2018). KSHV can infect multiple cell types, such as B-lymphocytes, lymphatic endothelial, vascular endothelial, and epithelial cells (Ganem, 2007). Latent herpesvirus infections and their reactivation during the immune suppression pose a significant risk in developing inflammatory diseases and cancer in humans (White et al., 2012). HSV-1 and HSV-2, and their association with cancer occurrence in humans are controversial (Flaitz et al., 1995; Jain, 2016). β herpesvirus HCMV is a cofactor involved in the etiology of inflammatory bowel diseases (IBDs), psoriatic plaques, rheumatoid arthritis, systemic lupus erythematosus (SLE), and Sjögrens syndrome (SS) (Britt, 2008). HCMV infection is prevalent in cancers of breast, colon, prostate, and brain (Cobbs et al., 2002; Harkins et al., 2002; Rahbar et al., 2003; Soderberg-Naucler, 2006; Herbein and Kumar, 2014). Clinical manifestation of primary infection of HHV-6 includes roseola infantum, a benign febrile exanthem of infancy in approximately 20% of the infected children (Stone et al., 2014). Unlike α- and β-herpesviruses, γ-herpesviruses EBV and KSHV are oncogenic as they can induce cancer in natural (human) or experimental hosts (Damania, 2004). EBV is primarily found in the tumor cells of Burkitt’s lymphoma (BL), lymphomas associated with immunosuppression, other non-Hodgkin’s lymphomas (NHL), Hodgkin’s disease, nasopharyngeal carcinoma (NPC), gastric adenocarcinoma, lymphoepithelioma-like carcinomas, post-transplant lymphoproliferative disorder (PTLD), nasal angiocentric T/NK-cell lymphoma, natural killer (NK)/T-cell lymphoma, and immunodeficiency-related leiomyosarcoma (Hsu and Glaser, 2000; Ambinder, 2003). γ-herpesvirus KSHV is etiologically associated with Kaposi’s sarcoma (KS), B cell lymphoproliferative primary effusion lymphoma (PEL), multicentric Castleman’s disease (MCD), and KICS (KSHV inflammatory cytokines syndrome) (Cesarman et al., 1995; Soulier et al., 1995; Karass et al., 2017).

Herpesviruses maintain a long-term evolutionary relationship with their host and stay latent in the host for their lifetime. Herpesviruses manipulate their host to hide, survive, replicate, produce new viral particles, and spread the viral infection to the uninfected host cells (Brinkmann et al., 2003; Wang et al., 2003; Feire et al., 2004; Wang et al., 2005; Sharma-Walia et al., 2010; Jung and Münz, 2015; Cavallin et al., 2018; Singh et al., 2018; Ayers et al., 2018; McNamara et al., 2019). Host cells are susceptible to productive or latent infection (Naranatt et al., 2004; Sharma-Walia et al., 2010; Grinde, 2013; Botto et al., 2017; Cavallin et al., 2018; Choi et al., 2018; McNamara et al., 2019). Host provides factors required for each step of herpesvirus complex life cycle that begins with its binding to the host cell and followed by multiple steps such as entry, uncoating, nucleic acid synthesis, gene expression, and viral protein synthesis (Naranatt et al., 2004; Sharma-Walia et al., 2010; Grinde, 2013; Botto et al., 2017; Cavallin et al., 2018; Choi et al., 2018; Singh et al., 2018; McNamara et al., 2019). In order to accomplish its survival and spread to uninfected cells, virus ends up utilizing host cell machinery for viral replication, gene transcription, infected cell division, and proliferation (Brinkmann et al., 2003; Naranatt et al., 2004; Sharma-Walia et al., 2010; Grinde, 2013; Jung and Münz, 2015; Botto et al., 2017; Cavallin et al., 2018; Choi et al., 2018; Singh et al., 2018; McNamara et al., 2019). Host factors include proteins of signaling cascades, transcription factors, cell survival pathways, angiogenic and growth factors, matrix metalloproteases, cell cycle kinases, cell death pathways, autophagy proteins, translation machinery, and inflammatory response pathways, immune evasion factors, chromatin remodeling, and metabolic pathways (Brinkmann et al., 2003; Naranatt et al., 2004; Sharma-Walia et al., 2010; Grinde, 2013; Jung and Münz, 2015; Botto et al., 2017; Ayers et al., 2018; Cavallin et al., 2018; Choi et al., 2018; Singh et al., 2018; McNamara et al., 2019).

**Figure 1:**
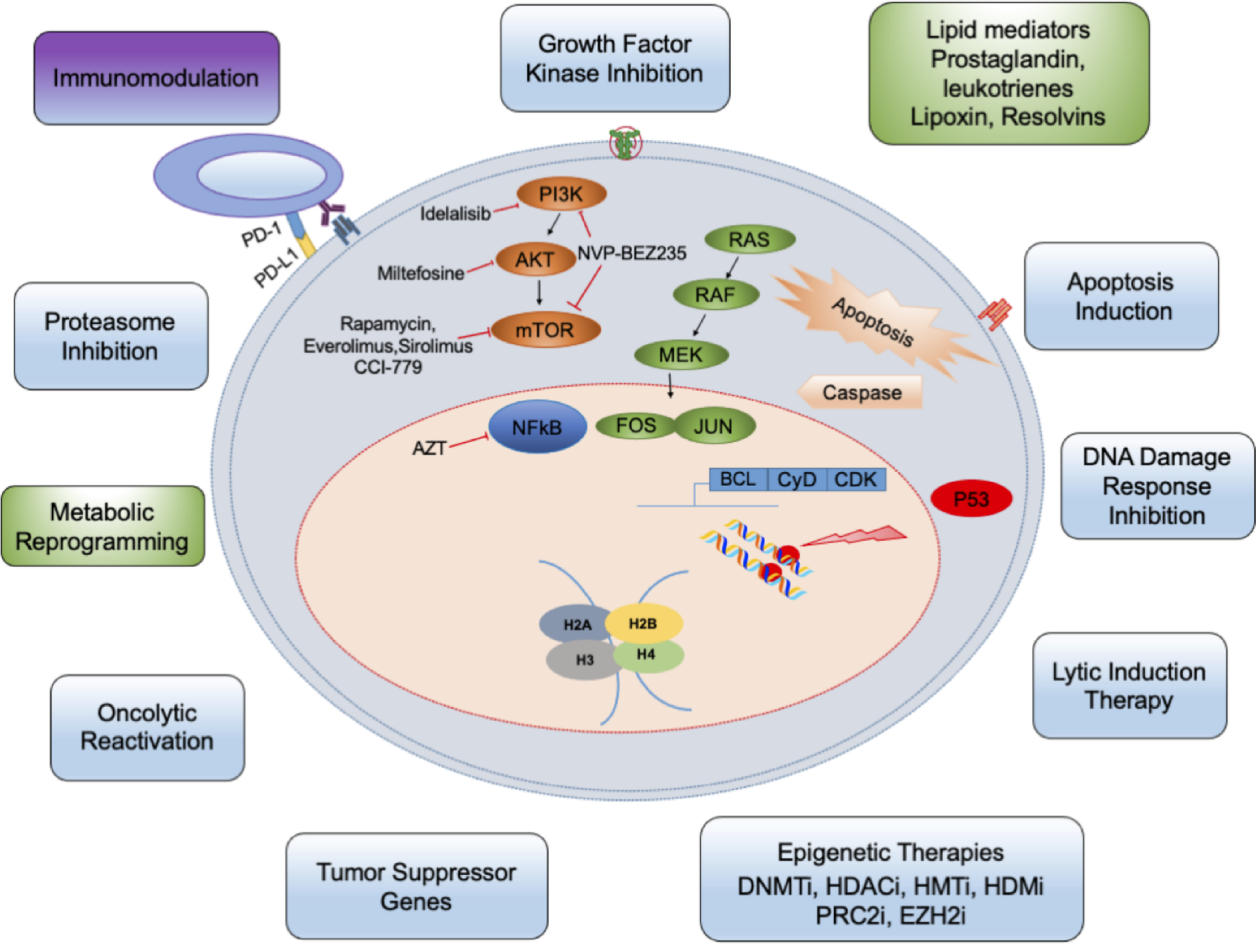
The targeted therapy principles against herpesvirus: Several metabolic and physiological processes including glycolysis, lipid synthesis, cholesterol metabolism are potentially altered in herpes infected cells (indicated by green boxes). Many cellular/signaling processes are also altered. The infection can be targeted by small-molecule inhibitors (blue) and other drugs. These inhibitors either target cyclin dependent cell cycle, epigenetic factors controlling viral life cycle or DNA damage, etc. Besides, immune cells (purple) and other cells of the tumor microenvironment can be targeted. DNMTi, DNMT inhibitors; HDACi, HDAC inhibitors; HMTi, HMT inhibitor; HDMi, HDM inhibitors; PRC2i, PRC2 inhibitors; EZH2i, EZH2 inhibitors.

Here, we focus only on the role of pro-inflammatory, anti-inflammatory, and resolution lipid metabolites of the arachidonic acid (AA) pathway in the lifecycle of herpesvirus infections. To date, cytotoxic systemic chemotherapies developed for non-virus-associated cancers are widely implemented for the treatment of herpesvirus associated cancers. These lesser effective treatment methods target DNA replication of all dividing cells and thus possess multiple side effects, particularly in immunocompromised patients and neonates. There is significant interest in developing new antiviral drugs targeting viral binding, entry, uncoating, viral nucleic acid synthesis, replication, gene expression, and viral protein synthesis. Here, we discuss recent development in the antiviral therapies targeting multistep lifecycle events of the viral life cycle, gene regulation, latency-lytic switch inducers, immunomodulators, and natural therapeutic compounds (**Figure 1**).

### Strategies to Combat Herpesviral Infection

#### Targeting Host Factors Involved in Multistep Herpesvirus Life Cycle

The herpesviruses enter host cells *via* a multistep process, which begins with a coordinated attachment of the virus to the host cell surface followed by interaction with specific binding and entry receptor(s), and subsequent induction of host signaling pathways to facilitate virus entry (Nemerow et al., 1985; Li et al., 1997; Haan et al., 2000; Speck et al., 2000; Spear and Longnecker, 2003; Wang et al., 2003; Feire et al., 2004; Wang et al., 2005; Chandran, 2010; Hahn et al., 2012; Hensler et al., 2014; Chen et al., 2019). Different viral envelope glycoproteins mediate herpesvirus entry into the host cell through either membrane fusion or receptor-mediated endocytosis (Nemerow et al., 1985; Tanner et al., 1987; Ligas and Johnson, 1988; Miller and Hutt-Fletcher, 1992; Turner et al., 1998; Muggeridge, 2000; Pertel, 2002; Feire et al., 2004; Naranatt et al., 2004; Compton, 2004; Avitabile et al., 2009; Chandran, 2010). Blocking herpesvirus binding, fusion, entry, and host signaling pathways is an attractive antiviral strategy to suppress viral infectivity (**Table 1**).

**Table 1:** Inhibitors targeting various stages of herpesvirus life cycle.

Drugs	Virus	Life cycle events
Compound SP-510-50 **PI3K inhibitor:** Idelalisib NVP-BEZ235 **Dermaseptins** **Endocytosis inhibitor:** Chlorpromazine **anti-gHgL antibodies:** CL40 and CL59 **Amantadine analog:** Tromantadine **Saturated fatty alcohol:** Docosanol	HSV-1 HSV-2, HCMV KSHV, EBV HSV-1, HSV-2, EBV KSHV, EBV EBV EBV HSV-1	Virus binding or entry inhibitors
**Epigenetic therapy**		
**DNMT inhibitors:** 5-azacytidine, RG108 **HDAC inhibitors:** MC1568, Sodium butyrate, Trichostatin A (TSA), Vorinostat (SAHA), Valproic Acid **HMT inhibitors:** DZNep (EZH2i) GSK126, GSK343 **HDM inhibitors:** ML342, DMOG, OG-L002, TCP	HSV-1, HSV-2, HCMV, VZV, EBV, KSHV HSV-1, HCMV HSV-1 HSV-1	Repression of viral immediate early gene expression. Production of infectious progeny in quiescently infected cells. Latency reversal Significant activation of the lytic transcriptional program in HCMV Blockage of lytic HSV-1 replication in latently infected ganglion explant model Repression of viral IE gene expression
**Nucleoside analogs**		
Trifluridine, Edoxudine, Brivudine, Cytarabine, Cidofovir, Acyclovir Ganciclovir, Penciclovir, Virdarabine, Indoxuridine **Non-nucleoside inhibitors:** Foscarnet PNU-183792 NSC 373989 **Helicase-primase inhibitor:** BAY 57-1293 T-0902611 BILS 179BS **Protein-protein interaction inhibitor** BILD1263	HSV-1, HSV-2, VZV, HCMV, EBV, HHV-6, HHV-7, KSHV HSV-1, HSV-2, VZV, HCMV HSV, VZV, HCMV KSHV HSV-1, HSV-2 HCMV HSV-1, HSV-2 HSV-1	DNA synthesis inhibitor. These drugs competitively stop the incorporation of dNTPs by the viral DNA polymerase and thereby end the elongation of viral DNA DNA synthesis inhibitors. Inhibit progression of the replication fork Inhibits viral replication
**Pyrazolo**		
Quinoline, Benzothiophene **Phosphorothioate oligonucleotides**	HSV-1 HCMV, EBV, KSHV	Gene expression
**Acridones, Thiourea inhibitors** **Phenylenediamine:** Sulfonamides **Ribosylbenzimidazoles**	HSV, EBV HCMV, KSHV HCMV	Assembly or egress

Herpesvirus binding and entry into the host are followed by uncoating viral DNA from the capsid, delivering viral genome to the nucleus, sequential transcription, and translation of viral immediate-early, early, late genes, and DNA replication. Nucleoside analogs have been tested for antiviral activity as these inhibit herpesviruses replication. Among all known inhibitors are nucleoside analogs, non-nucleoside analogs, helicase-primase inhibitor, and protein-protein interaction inhibitors that stop DNA replication, transcription, and translation (**Table 1**). Acyclovir, a synthetic nucleoside analog, has been efficiently used against HSV-1/2 and VZV-associated disease (**Table 1**). Ganciclovir, cidofovir, foscarnet, and letermovir have also been used successfully in patients with herpesvirus infection (**Table 1**). Inhibitors blocking virion assembly and egress of newly assembled herpesvirus particles from infected cells can be used effectively to treat herpes virus infections (**Table 1**). Cyclin-dependent kinases inhibitors that prevent proliferation in cancer cells are also used against herpesviruses (**Table 2**). These cellular protein kinase inhibitors play a crucial role in managing ER stress and active unfolded protein response (UPR) associated with various cancers and viral malignancies (Asha and Sharma-Walia, 2018).

**Table 2:** Cellular protein kinase tested for anti-herpesvirus associated cancers.

Drug	Virus	Primary targets	Ref
Chk2 inhibitor II	HSV	Chk2	(Alekseev et al., 2015)
Roscovitine (seliciclib)	HSV, VZV, HCMV and EBV	CDKs	(Schang et al., 2002; Kudoh et al., 2004; Taylor et al., 2004; Shin et al., 2008)
Purvalanol A	HSV, VZV and EBV		(Schang et al., 2002; Kudoh et al., 2004; Moffat et al., 2004)
Olomoucine II	HSV and HCMV		(Moffat et al., 2004)
Indirubin-3’-monoxime	HCMV	GSK3β	(Hertel et al., 2007)
Torin1	HSV and HCMV	mTOR	(Li and Hayward, 2013)
Everolimus	HCMV, EBV		(Poglitsch et al., 2012; Rittà et al., 2015)
Letermovir	HCMV	Terminus inhibitor	(Gerna et al., 2019)
Maribavir	HCMV	UL97 viral protein kinase	(Papanicolaou et al., 2019)
KU55933	HSV, HCMV and EBV	Ataxia telangiectasia mutated (ATM)	(Hagemeier et al., 2012; Costa et al., 2013; Alekseev et al., 2014)
TBB	HSV-1 and EBV	Casein kinase 2 (CK2)	(Medina-Palazon et al., 2007; Smith et al., 2011)
DMAT			
TMCB			
SP600125	VZV	c-Jun N-terminal kinase (JNK)	(Zapata et al., 2007)
Gleevec (imatinib mesylate)	HCMV, KSHV	Inhibits multiple tyrosine kinases	(Soroceanu et al., 2008; Koon et al., 2014)
Sorafenib (BAY 43-9006)		PDGFR and Raf kinases	(Michaelis et al., 2011)
STO-609		Calmodulin-dependent kinase kinase (CaMKK)	(McArdle et al., 2011; McArdle et al., 2012)
LY294002		PI3K	(Johnson et al., 2001a)
U-0126	HCMV and EBV	MEK1/2	(Johnson et al., 2001b; Goswami et al., 2012)
Flavopiridol (alvocidib)	EBV	CDKs	(Goswami et al., 2012)
Kenpaullone		GSK3β, LcK	(Goswami et al., 2012)
Dasatinib		Inhibits multiple tyrosine kinases	(Chakraborty et al., 2012; Goswami et al., 2012; Hahn et al., 2012)
K252A			
BAY 11-7082		Inhibitor of κB kinase (IKK)	(Goswami et al., 2012)
Wortmannin		Phosphatidylinositol-3-kinase (PI3K)	(Goswami et al., 2012)
PCI-32765		Bruton’s tyrosine kinase (Btk)	(Zocchi et al., 2018)
MLN8237		Aurora A kinase	(Iankov et al., 2015)
CCI-779 (temsirolimus)		mTOR	(Kawada et al., 2014)
BI-D1870		p90 ribosomal S6 kinases (RSKs)	(Kuang et al., 2008)
SU5416		VEGFR tyrosine kinase	(Aoki and Tosato, 2004)

### Epigenetic Targeted Therapy

Recent studies focusing on herpesvirus infections identifying viral proteins regulating histone-modifying enzymes and regulating the transcriptional machinery have brought revolution in the development of antiviral epigenetic modifiers (Kristie, 2012) (**Table 1**). Histone deacetylation and methylation are associated with condensed chromatin (heterochromatin) and accompany transcription inhibition, whereas histone acetylation and demethylation do the reverse. For many of herpesviruses, viral DNA associates itself with histones as soon as it enters the cell, making it a vulnerable candidate to epigenetic modification (Reeves, 2011). These modifications are basic cellular defense mechanisms against viral gene expression and to prevent abortive infection. Eventually, at the onset of the lytic cycle during stress or treatment with histone modifier, the viral genome opens up from its repressed heterochromatin state to initiate the expression of immediate early lytic genes followed by productive infection (Woodhall et al., 2006). Arsenal of histone modifiers have been identified over the last decade, which play an important role(s) throughout the different viral life cycle stages (Kristie, 2012). Histone modifiers include writers (the enzymes that make the chemical modifications), readers (proteins that detect and respond to the chemical modifications), and erasers (enzymes that remove these chemical groups). DNA methyltransferases (DNMTs), histone methyltransferases (HMTs), and histone acetyltransferases (HATs) are writers. Readers include chromatin remodeler complex SWItch/Sucrose Non-Fermentable (SWI/SNF), the bromodomain (BRD), and extra terminal domain family of adaptor proteins (BET). Erasers are DNA-demethylating enzyme histone demethylase (HDM) and histone deacetylases (HDACs). Epigenetic therapy (inhibitors of DNMTs, HDAC, HMT, chromatin-modifying complex; The polycomb repressive complex 2 or PRC2, and enhancer of zeste homolog 2 or EZH2) has been well studied and demonstrated success in HSV-1, HSV-2, HCMV, EBV, and KSHV latency and viral HCMV-HSV, HIV-HCV, and HIV-HBV viral co infections (**Table 1**) (Schultz et al., 2002; Bain et al., 2003; Gwack et al., 2003; Wright et al., 2005; Rosenfeld et al., 2006; Abraham and Kulesza, 2013; Kumar and Herbein, 2014; Torres and Tang, 2014; Su et al., 2017; Hopcraft et al., 2018). Backed up by favorable results from both cell culture labs and mouse models, epigenetic drug candidates are clinically evaluated as promising antivirals. Despite the roadblocks in designing specific epigenome modifying drugs with few off-target effects, there are ongoing clinical trials to enhance safety using improved preclinical models. The combination of valganciclovir with Tractinostat (VRx-3996) is now being examined in EBV-related lymphoid malignancies. HDAC inhibitors such as benzamide MS275 or sodium butyrate (**Table 1**) might induce lytic gene expression and act as stimulators to antivirals like ganciclovir used for the cure of EBV-associated lymphomas (Ghosh et al., 2012). In another clinical study, DNMT inhibitor azacytidine (**Table 1**) initiated viral gene re-expression in EBV linked tumors (Chan et al., 2004). While these inhibitors are promising candidates as the therapeutics to control of herpesviral infections (**Figure 1**), the possibilities of reactivation and status of co-infection with other viruses must be meticulously examined (Ritchie et al., 2009). For example, the usage of suberoylanilide hydroxamic acid (SAHA) or trichostatin A (TSA) (**Table 1**) provoked myocarditis through Coxsackievirus B3-induced myocardial apoptosis (Zhou et al., 2015).

An *in vivo* study showed that bortezomib (Btz) usage, a proteasome inhibitor, improved the survival in an immune-compromised xenograft mouse model of PEL that was treated with doxorubicin alone (Sarosiek et al., 2010). A combination of Btz and HDAC inhibitor, SAHA could effectively reactivate KSHV, thereby inducing PEL cell death and increasing survival in PEL-bearing mice, and strongly advocates using the proteasome/HDAC inhibitor combination therapy in PEL (Bhatt et al., 2013).

### Lytic Cycle Induction and Combination Therapies

Latency intervention is one of the well-known strategies to target herpes infection and control herpes virus associated cancers. Among all known herpesviruses, EBV is the only virus for which proteins associated with maintenance of latency have been best characterized and tested for the anti-latency approach. There have been attempts to target EBV nuclear antigen (EBNA1) and latent membrane protein 1 (LMP1) using antisense oligonucleotides or adenovirus vector-delivered ribozymes (Li et al., 2010). Cellular signaling kinase associated with the LMP2A pathway has been targeted to cure EBV infection (Li et al., 2010). However, more effective strategies against the virus could be unmasking of latently infected cells by inducing lytic reactivation and then explicitly targeting viral DNA replication. In recent work Rauwel et al., 2015, suggested that knocking down transcriptional corepressor Krüppel-associated Box-associated protein 1 (KAP1) or induction of KAP1 phosphorylation can force HCMV out of latency, and this process can be made possible by activating NF-kB with TNF-α. These results suggest new approaches both to limit HCMV infection and to eliminate the virus from organ transplants (Rauwel et al., 2015). In a similar study authors suggested that Chloroquine employs ataxia telangiectasia mutated (ATM) to phosphorylate the KAP1/TRIM28 at serine 824 to facilitate repair of double-stranded breaks in heterochromatin and triggers EBV replication (Li et al., 2017). Further studies proved that EBNA1 and LMP1 associated sumoylation plays a crucial role in the maintenance of EBV latency through KAP1 (Bentz et al., 2015). Therefore, EBNA1^SIM^ motif can play a potential drug target against EBV-associated cancers (Wang et al., 2020). KSHV latency-associated nuclear antigen (LANA) interacts with the host protein, KAP1, and represses lytic gene expression to facilitate the establishment of KSHV latency (Sun et al., 2014; Zhang et al., 2014). Further studies proved that the LANA has an exclusive SUMO-interacting motif (LANA^SIM^), which plays an indispensable role and thus can play a potential drug target against KSHV-associated cancers (Cai et al., 2013).

Reactivation from latency is vital for developing therapies to fight or eliminate herpes-associated cancers, and this strategy is successful against herpes infection in HIV-positive patients. One of the most talked-about methods has been “*lytic induction therapy*”. This therapy is based on the theory of synergistic usage of antiviral drugs and the stimulation of viral reactivation. This approach was successful against PEL growth in mouse xenograft models (Feng et al., 2004; Fu et al., 2008; Bhatt et al., 2013; Zhou et al., 2017). This method has been extensively investigated for KSHV and EBV. *Ex vivo* studies have established romidepsin as an effective inhibitor that works against lymphoproliferative diseases (Smolewski and Robak, 2017) and is a better agent for viral reactivation (Wei et al., 2014) than other HDAC inhibitors. Likewise, a recent study from our lab has shown anti-inflammatory lipoxin A4 (LXA4) as a promising candidate for lytic induction therapy (Asha et al., 2020). LXA4 treatment regulates KSHV reactivation and life cycle through chromatin modification (Asha et al., 2020) and the host’s hedgehog signaling pathway (Asha et al., 2020).

The most recent strategies chosen to treat the herpesvirus is based on the concurrent induction of oncolysis by viral replication and reassertion of an immune response to viral lytic cycle antigens. For example, Oncolytic HSV (G47Δ) has shown its improved efficacy against NPC (Wang et al., 2011), glioma, breast cancer (Liu et al., 2005), and other fatalities and thus can be used in combination with immunotherapy and chemotherapy to treat various malignancies. Such combinational strategies can also be used against EBV-associated lymphoproliferative diseases. Ganciclovir (GCV) is a nucleoside analog antiviral drug that facilitates the killing of EBV-positive cancer cells when given lytic inducers. BGLF4, an EBV lytic protein kinase expressed during viral reactivation, can change GCV into its cytotoxic state (Feng et al., 2002), which gets integrated into viral and host DNA. Cytotoxic GCV induces termination of premature DNA to kill EBV-associated malignancies and causes apoptosis of the host cells (**Table 1**) (Feng et al., 2004; Feng and Kenney, 2006; Wang et al., 2010). Similarly, [125I]2′-fluoro-2′-deoxy-beta-D-5-iodouracilarabinofuranoside ([125I]FIAU) has been used against lytically induced EBV-positive BL cells (Fu et al., 2008). In NPC patients, the usage of valproic acid (**Table 1**) and gemcitabine as lytic inducers has shown effective clinical response generating moderate momentary toxicity (Wildeman et al., 2012). In a recent study, derivatives from biologically active alkaloid tetrahydrocarboline were found to reactivate EBV lytic markers Zta (ZEB replication activator, the product of BZLF1) and Early Diffuse Protein in all EBV-positive cell lines irrespective of their type of latency. Two of these derivatives have EC50 values in the range of 150–170 nM and showed low toxicity to EBV-negative cells. When combined with GCV, these small molecules were selectively cytotoxic to EBV-positive cells (Tikhmyanova et al., 2014).

### Therapeutic Compounds From Natural Extracts

Much research focuses on identifying new therapeutic compounds obtained from natural plant extracts with inhibitory activity against herpesviruses (**Table 3**). These natural plant extracts have proved effective against different stages of viral infection including viral binding/entry, replication, and release. Extracts from Allium sativum has proved effective against viral adsorption and penetration (Rouf et al., 2020). *Epigallocatechin-3-gallate (EGCG)*, the active ingredient in green tea, has potential antiviral activity against HSV-1 (de Oliveira et al., 2013) and lytic infection of EBV (Chang et al., 2003; Liu et al., 2013) and KSHV (Xie et al., 2020). Herbal extract from *Rhus javanica*, a medicinal herb has two major anti-HSV compounds called moronic acid and betulonic acid (Kurokawa et al., 1999). Moronic acid inhibits EBV lytic cycle (Chang et al., 2010). *Protoapigenone*, a naturally occurring group of flavonoid compound obtained from *Thelypteris torresiana*, inhibits EBV lytic replication (Tung et al., 2011). The diterpenoid andrographolide present in *Andrographis paniculata*, a medicinal plant, has proven effective against active EBV virions (Lin et al., 2008). The anti-inflammatory and anti-immunostimulatory activities of the diterpenoid andrographolide were effective against inflammatory diseases, bacterial and viral infections (Wiart et al., 2005; Aromdee et al., 2011; Uttekar et al., 2012). *Emodin*, a significant component from *Polygonum cuspidatum*< intervenes with the early steps of the EBV replication cycle in a dose-dependent manner (Yiu et al., 2014). Emodin inhibits the activation of ERK, MAPK, and JNK signaling, and affect the activation of the promoters stimulated by transcription factor activator protein-1 (AP-1) and activating transcription factor 1 (ATF1) (Lee et al., 2006; Lin et al., 2007; Li et al., 2013). Lignans obtained from *Saururus chinensis* inhibits NF-kB (Hwang et al., 2003) and HIV protease (Lee et al., 2010). *Manassantin B*, another lignans fraction isolated from *Saururus chinensis*, works effectively against active EBV infection at significantly low toxicity (CC50 > 200 µM) dose (Cui et al., 2014). Likewise, *Angelicin*, a natural compound found in the roots of *Angelica archangelica*, inhibits the autoactivation of the EBV and KSHV RTA promoter and consequential interferes with the initial lytic viral replication (Cho et al., 2013) (**Table 3**). Through a high-throughput screening of characterized compounds, Gruffaz et al. showed that *cytarabine*, an FDA-approved compound, induced regression of PEL tumors in a xenograft mouse model. Interestingly, cytarabine degraded KSHV latency protein LANA-1, which is required for PEL cell survival (Gruffaz et al., 2018). Furthermore, cytarabine inhibited KSHV lytic replication program, preventing virion production. These findings suggest cytarabine to be a novel therapeutic agent for treating PEL as well as for eliminating KSHV persistent infection (Gruffaz et al., 2018) (**Table 3**). Cambogin, a bioactive natural product isolated from the *Garcinia genus* when used at nanomolar concentration, could reduce the PEL tumor in xenograft mouse model (Ding et al., 2019). Dong-Eun Kim et al. showed that treatment with *Euphorbia pekinensis* extract led to a selective oncolytic effect on EBV-positive gastric carcinoma cells, SNU-719 (Kim et al., 2018). *E. pekinensis*’s ability to induce lytic activity was mediated by PKC and MEK signaling (Kim et al., 2018) (**Table 3**). Likewise, the combination of FDA-approved drug ingenol-3-angelate (PEP005) with a BRD and BET protein inhibitor (JQ1) induced KSHV lytic replication and reduced IL6 production in the PEL model (Zhou et al., 2017). The combination of drug PEP005 and JQ1 inhibits PEL growth efficiently and interrupts tumor growth in a PEL xenograft tumor model (Zhou et al., 2017). PEP005 activates NF-κB pathway that primes the increased occupancy of RNA polymerase II onto the KSHV genome, thereby reactivating KSHV (Zhou et al., 2017). Allicin, and glycyrrhizic acid (GA) obtained from garlic and licorice respectively, had antiviral effects against latent KSHV infection (Xie et al., 2020) (**Table 3**).

**Table 3:** Natural extracts effective against Herpesvirus infection.

Plant	Active compound	Mode of action
*Spirulina platensis*	Sulfated polysaccharides Calcium spirulan	Viral entry (HSV-1, HCMV, VZV) Inhibition of cytopathic effect blocking HCMV attachment and penetration into host cells
*Allium sativum*	Organosulfur compound	Interferes with HSV adsorption and penetration
*Green tea*	*Epigallocatechin-3-gallate (EGCG)*	Anti-HSV-1 Inhibits lytic infection of EBV and KSHV
*Rhus javanica*	Moronic acid and Betulonic acid	Inhibits lytic infection of EBV
*Thelypteris torresiana*	*Protoapigenone*; flavonoid compound	Inhibits lytic infection of EBV
*Andrographis paniculata*	andrographolide	Inhibits active EBV virions
*Polygonum cuspidatum*	*Emodin*	Inhibits early steps of the EBV replication cycle
*Saururus chinensis*	Lignans *Manassantin B*	Inhibits NF-kB against active EBV infection
*Angelica archangelica*	*Angelicin*	Anti-HSV-1 Interferes with the initial lytic KSHV replication
*Marine natural product*	*Cytarabine*	Regression of PEL tumors in a xenograft mouse model. Degrades LANA-1, inhibit latent and lytic replication
*Garcinia*	Cambogin	Regression of PEL tumors in a xenograft mouse model
*Euphorbia pekinensis*	Euphorbia extracts	Oncolytic effect on EBV-positive gastric carcinoma cells
*Euphorbia peplus*	PEP005	Inhibits PEL tumors in a xenograft model
*Allium sativum*	Allicin	Interferes with HCMV replication Inhibits latent KSHV

### Immune Modulation

Extensive modification of the host immune system by herpesviruses has opened up new prospects for immunotherapy as clinical intervention to effectively treat herpesvirus-associated malignancies. Recent studies have identified immune cells and blood cells as target of herpes infection (Hill et al., 2010; Quinn et al., 2016; Jones et al., 2019). Successful cancer immunotherapy involves immune effectors of both the innate (innate lymphoid, NK, T, and B cells) and adaptive immune systems to develop anticancer immunity. Immunotherapy includes cancer vaccines to prime and expand tumor-specific T cells with potent antitumor activity and immune checkpoint blockers to abrogate negative inhibitory signals that diminish T-cell activation (Vanpouille-Box et al., 2017). Inhibitory molecules overexpressed in many tumors that facilitate immune escape include cytotoxic T lymphocyte associated protein 4 (CTLA4), programmed cell death 1 (PDCD1 or PD1), and PD1 ligand CD274 (PD-L1) (Jinesh et al., 2017). Anti-PD1/PD-L1 is the most promising immunotherapy currently (**Figure 1**).

Opportunistic infection is still the main reason for mortality in allogeneic stem cell transplant recipients with active extensive chronic graft-*versus*-host disease. The toxicity of prolonged and recurring antiviral treatment along with occasional drug resistance account for major limitation to therapy. For the effective treatment, successful propagation of virus-specific donor-derived CD8+ CTLs is essential. CTLs are effective against VZV, HCMV, and HHV-6 infections in the immunocompromised patients (Kapp et al., 2007; Hill et al., 2010; Gerdemann et al., 2013; Becerra et al., 2014; Quinn et al., 2016; Jones et al., 2019). EBV-specific CTL immunotherapy has raised hopes of treating NPC effectively (Jain et al., 2016; Cao, 2017). EBV proteins such as LMP1, LMP2, and EBNA1 are used to develop vaccines that can be used as adjuvant therapy to avoid NPC relapse (Jain et al., 2016; Taylor and Steven, 2016). KSHV infected monocytes express high levels of PD-L1 (Host et al., 2017). NK cells obtained from KS patients frequently display higher levels of PD1 (Beldi-Ferchiou et al., 2016). Usage of immune checkpoint blockades such as nivolumab or pembrolizumab showed effective treatment against KS infection in HIV patients (Galanina et al., 2018; Uldrick et al., 2019). Pembrolizumab has successfully made through phase I clinical trial for patients living with HIV and KS and is in its phase II trial against KSHV (Uldrick et al., 2019). Aiming two distinct T cell inhibitory proteins simultaneously may end up in a more significant T cell function. Based on this theory, combination therapy of ipilimumab and nivolumab has been tried on classical KS patients (Lingel and Brunner-Weinzierl, 2019). The FDA approved Pomalidomide, after the phase I/II clinical is used in a new clinical trial in the endemic population of KS (Polizzotto et al., 2016). Pomalidomide treatment could reestablish MHC-I expression during the lytic replication and restore T cell costimulators (B7-2) in PEL cells (Davis et al., 2017). Blocking PD-L1 can effectively enhance virus specific CD8+ T cell effector functions (Jones et al., 2019).

In HSV-1 and HSV-2 infection, both humoral and cellular immune responses are specifically directed towards the surface glycoproteins gB and gD (Cairns et al., 2014). Animal studies on HSV-1 and HSV-2 demonstrated that humanized antibodies focused on these receptor proteins are prophylactically and therapeutically beneficial (Baron et al., 1976; Bravo et al., 1996). Rituximab, an anti-CD20 antibody, provides clinical benefit by inducing B cell death upon binding and is efficient against PEL and KSHV-MCD (Hoffmann et al., 2011; Kim et al., 2014). Ongoing clinical trials also use combinations of lenalidomide, chemotherapy and rituximab. Clinical trial of anti-IL6 antibodies was carried against MCD (Yu et al., 2017).

As an alternative, attempted antivirals can aim at controlling periodic infections by boosting immunity. For instance, resiquimod controls the innate immune response to prevent genital herpes infection (Govindan, 2014). Oncolytic virotherapy using genetically engineered replication competent viruses to destroy cancers is also an emerging treatment modality for various malignancies. The combination of oncolytic virotherapy with other possible immune-based therapies would prove beneficial for malignancies linked to viral infections (Zamarin and Wolchok, 2014).

### Targeting Inflammatory Membrane Lipid Pathways

**Figure 2:**
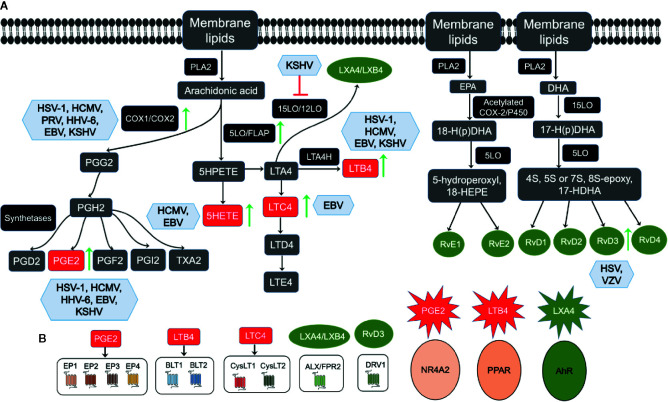
Overview of membrane lipids metabolism and herpesvirus infections: **(A)** Membrane lipids move to the cytoplasm by the activation of calcium-dependent cytosolic phospholipase A2 (cPLA2) to form Arachidonic acid (AA), eicosapentaenoic acid (EPA), and docosahexaenoic acid (DHA). AA’s movement to the cytoplasm by cPLA2 liberates free AA, which enzymatically metabolizes to eicosanoids *via* three major pathways, such as the cytochrome P450, cyclooxygenase (COX), and 5-lipoxygenase (5LO). AA is converted to the intermediate prostaglandin H2 (PGH2), which is then metabolized to prostaglandins (PGs), such as PGE2, PGF2, PGD2, and PGI2, and thromboxane TXA2 by specific synthetases. In the 5LO pathway, AA is metabolized by 5LO and 5-lipoxygenase activating protein (FLAP) to 5-HPETE, which then forms leukotriene A4 (LTA4). LTA4 is consequently transformed into LTB4 by LTA4 hydrolase or glutathione to LTC4 by LTC4-synthase and glutathione-S-transferase. LTC4 forms LTD4 and LTE4 *via* ubiquitous enzymes. LTA4 can be catalyzed by 15LO/12LO enzymes to form anti-inflammatory lipoxins as LXA4 and LXB4. The resolvins family is divided primarily into two groups, E-series resolvin, and D-series resolvin, based on their parent omega-3 PUFA. For E-series resolvin biosynthesis, EPA is the substrate for acetylated COX or cytochrome P450 enzymes, giving rise to 18-hydroperoxide (18-H(p)DHA), which then forms either resolvin (Rv) E1 or RvE2 by 5LO. Furthermore, DHA is the substrate for 15LO, giving rise to a 17-hydroperoxide (17-H(p)DHA), which is subsequently converted to D-series resolvins (RvD1, RvD2, RvD3, RvD4). Viral infections regulating the AA pathway enzymes or metabolites are shown in blue boxes, upregulation is shown by green arrows, and inhibition by red.** (B)** PGE2, LTB4, LTC4, LXA4/LXB4. RvD3 governs its downstream functions *via* interaction with its respective receptor, as indicated.

Herpesvirus infections create a unique inflammatory microenvironment conducive to its latency and survival in the host. A few components of this microenvironment milieu are cytokines, chemokines, growth factors, and lipid pathway metabolites. Recent studies revealed that inflammatory cytokines activate phospholipases to induce the release of polyunsaturated fatty acids (PUFAs) from the cell membrane phospholipids. PUFAs generate bioactive lipids that augment the anti-cancer action of immunotherapy, prevent cytokine storm, and play an essential role in eliminating cancer cells. Herpesviruses utilize a PUFA called Arachidonic acid (AA), which is present in the phospholipids of the cell membrane and its downstream metabolites and receptors to maintain their successful life cycle in the host (**Figures 2A, B**) (Janelle et al., 2002; Konson et al., 2004; Gebhardt et al., 2005; Hooks et al., 2006; Chandrasekharan et al., 2016; Gandhi et al., 2017). AA is transformed into various bioactive lipid mediators to regulate inflammatory networks in the host cell (Samuelsson, 1991). AA is released from the membrane by the action of the enzyme phospholipase A2 (PLA2) (**Figure 2**). AA is metabolized by the pro-inflammatory cyclooxygenase (COX) and lipoxygenase (LO) pathways to produce eicosanoids (all-cis-5, 8, 11, 14-eicosatetraenoic acid) such as prostaglandins (PGs) and leukotrienes (LTs) (Fanning and Boyce, 2013; Chandrasekharan et al., 2016) (**Figure 2A**).

#### Cyclooxygenase Pathway

COX1 and COX2 are two distinct isoforms of the COX enzyme COX1 expression, where COX1 is constitutive and generates prostaglandins for normal physiological functions, and COX-2 is inducible in cancers and viral infections (Vane and Botting, 1998). AA converts to the intermediate prostaglandin H2 (PGH2), which then uses specific synthetases to synthesize PGE2, PGF2, PGD2, and PGI2, and thromboxane. Pro-inflammatory PGE2 mediates its actions by its interaction with the rhodopsin superfamily of serpentine receptors (G-protein-coupled seven-transmembrane-domain receptors), also called E‐type prostanoids (EP) receptors such as EP1, EP2, EP3, and EP4 (**Figure 2B**). Functional EP receptors have been localized on the plasma membrane and nuclear membranes in the perinuclear region of a variety of cell types and tissues (Bhattacharya et al., 1999). PGE2 has also been shown to induce the expression and activity of a nuclear receptor superfamily member nuclear factor NR4A2 (Nurr1) in colorectal carcinoma cells by PGE2 (Holla et al., 2006).

HSV-1 recurrent infection reactivates the latent virus in the central nervous system. The reactivation of the virus promotes the onset of painful lesions in peripheral tissues and presents a compelling clinical health issue. The reactivation process reprograms the host gene expression and transcription factors (Shimeld et al., 2001), and the inflammatory cyclooxygenase-2 (COX2) pathway is one among those (Gebhardt et al., 2005). HSV thymidine kinase (HSV-tk) gene transduction augments tumor growth rate, COX2 protein, and prostaglandin E2 (PGE2) release in murine colon cancer cells (Konson et al., 2004). Increased level of PGE2 promoted the development of resistance against antiviral GCV treatment in HSV-tk related tumor development (Konson et al., 2004). COX2 inhibitor nimesulide treatment inhibited the growth rate of HSV-tk-transduced murine tumors (Konson et al., 2004). Oral therapy with COX2 selective inhibitor, Celecoxib reduced viral reactivation in the trigeminal ganglia, suppressed virus shedding when given prophylactically by the gastrointestinal route in mouse models (Gebhardt et al., 2005). HSV-1 reactivation and nerve inflammation are involved in vertigo-related vestibular pathogenesis. Non-steroidal anti-inflammatory drugs (NSAIDs), including indomethacin and Celecoxib, are frequently used to suppress reactivation of HSV-1 (Liu et al., 2014). However, whether NSAIDs can affect the reactivation of HSV-1 in vestibular ganglions is not certain. Future studies are required to confirm the effects of NSAIDs on the HSV-1 life cycle (Liu et al., 2014). Celecoxib treatment could significantly suppress HSV-1 reactivation, reduce the numbers of corneas and ganglia containing the infectious virus, and placebo treatment in mice. These experiments strengthen the possibility of using COX2 inhibitors to prevent HSV-1 reactivation in high-risk patients by drug prophylaxis (Gebhardt et al., 2005). Intraperitoneal therapeutic and oral prophylactic plus treatments of a non-specific inhibitor of cyclooxygenases such as acetylsalicylic acid (ASA) could inhibit ocular reactivation shedding of HSV-1 in tears in mice model (Gebhardt et al., 2004). These findings implicate that using non-toxic inhibitors of prostaglandin synthesis may be useful in humans suffering from HSV-1 complications (Gebhardt et al., 2004). Pseudorabies virus (PRV), an α-herpesvirus distantly related to HSV-1, increases COX2 mRNA and protein level in PRV infected rat embryonic fibroblast cells (Ray et al., 2004). HCMV establishes lifelong persistence and latent infection following primary exposure and is a common cause of opportunistic infections and subsequent morbidity and mortality in immunocompromised patients. CMV immediate early (IE) protein and COX2 proteins were identified in CMV infected human retinal pigment epithelial (RPE) cells in retinal tissue sections from patients with CMV retinitis. Induction in COX2 mRNA, the protein was also accompanied by increased PGE1 and PGE2 levels in HCMV infected human RPE cell cultures. The induction of COX2 and PGE2 during retinal HCMV infection was suggested to augment virus replication HCMV retinitis (Hooks et al., 2006).

HCMV encodes the constitutively active chemokine receptor US28, which induces an oncogenic phenotype both *in vitro* and *in vivo via* induction of COX2 expression. US28 stimulates COX2 expression *via* activation of NF-kB. Targeting COX2 *in vivo* with Celecoxib significantly delayed the onset of tumor formation in nude mice injected with US28-transfected NIH-3T3 cells. Celecoxib treatment reduced the subsequent growth of the tumor by downregulating the US28-induced angiogenic activity (Maussang et al., 2009). mCMV (mouse cytomegalovirus) induced COX2 has been reported to induce ERK phosphorylation necessary for viral pathogenesis (Melnick et al., 2011).

HHV-6 infection induces COX2 gene expression and PGE2 synthesis within a few hours of infection of monocytes/macrophages. HHV-6 immediate-early protein 2 was discovered as a modulator of COX2 gene expression in monocytes/macrophages, and the addition of PGE2 could increase HHV-6 replication (Janelle et al., 2002).

COX2 pathway has been extensively studied in the EBV mediated tumorigenesis, and EBV induced proliferation of B lymphocytes and COX2, PGE2, and PGE2 receptors EP 1-4 are frequently over-expressed in EBV positive cancer cells in people with chronic inflammatory conditions, Burkitt’s lymphoma, and NPC (Gandhi et al., 2015). Upregulated COX2 has been shown to modulate EBV latency through its downstream effector PGE2 (Gandhi et al., 2015). EBV has been shown to suppress the biosynthesis of PGE2 in monocytes *via* inhibition of the inducible COX2 isoform expression both at the transcriptional and translational levels, not altering the gene expression of the constitutive COX1 isoform. This regulation serves as EBV’s strategy to evade immune surveillance. The inhibition of PGE2 biosynthesis was relieved in the presence of an inhibitor of herpesviruses DNA polymerase, demonstrating that viral replication and viral proteins were involved in this process (Savard et al., 2000).

COX2, its lipid metabolite PGE2, PGE2 receptors, or eicosanoid receptors (EP1-4) have been widely studied in KSHV associated diseases such as endothelial cell tumor KS and PEL (George Paul et al., 2010; Sharma-Walia et al., 2010; Paul et al., 2011; Paul et al., 2013). Therapeutic potential of non-steroidal anti-inflammatory drugs (NSAIDs) targeting COX-2, 5LO pathways, PGE2 receptor (EP receptor) antagonists blocking LTB4 secretion has been tested in the vascular malignancy KS and B cell lymphoproliferative disease PEL (George Paul et al., 2010; Paul et al., 2011; Paul et al., 2013; Sharma-Walia et al., 2014). Besides herpesviruses, COX2 is one of the critical mediators of inflammation in response to other viral infections such as the dengue virus (DENV), which utilizes it in replication (Lin et al., 2017).

#### Lipoxygenase Pathway

The Lipoxygenase pathway consists of 5LO, 8LO, 12LO, and 15LO enzymes and their products, leukotrienes (LTs), including LTA4, an unstable intermediate, LTB4, LTC4, LTD4, and LTE4 (Smith and Fitzpatrick, 1991). Leukotrienes are potent pro-inflammatory lipid mediators that play a central role in cardiovascular diseases, including arteriosclerosis, myocardial infarction, stroke, and have also been tested in viral infections related to inflammation. Leukotriene LTB4 is a highly chemotactic lipid mediator that triggers adherence to the endothelium, activates, and recruit leukocytes to the site of injury, and plays a pathogenic role in inflammatory diseases. BLT1 and BLT2 receptors can recognize LTB4 (**Figure 2B**) (Tager and Luster, 2003). LTB4 is a physiologically relevant endogenous ligand for the peroxisome proliferator-activated receptor alpha (PPARα) (Fiedler et al., 2001; Narala et al., 2010). LTC4 binds G-protein coupled receptors (GPCRs) called CysLT1 and CysLT2, mediating calcium flux, and activate signaling cascades (**Figure 2B**) (Evans, 2002).

Selective lipoxygenase inhibitor, TEI-1338, has been tested to inhibit HSV-1 infection *in vivo* with reduced protein leakage into aqueous humor in the rabbit corneal infection model (Bazan, 1988; Limberg et al., 1988). TEI-1338 has also been recommended to treat human herpes keratitis (Bazan, 1988; Limberg et al., 1988). In the context of the lipoxygenase pathway, the chemotactic leukotriene LTB4, in the presence of Ca2+, could significantly augment the killing of HSV-1 infected cells by enhancing target cell recognition by cytotoxic effector cells and subsequently by expanding their lytic efficiency (Gagnon et al., 1987).

HCMV infection of human vascular smooth muscle cells (SMCs) increases 5LO mRNA levels enabling them to synthesize bioactive LTB4 (Qiu et al., 2008). HCMV-infected vascular SMCs expression of 5LO protein and leukotriene production contributes to local inflammation and pathogenesis (Qiu et al., 2008). HCMV *ex vivo* infection in human placentae and umbilical vein endothelial cells (HUVEC) demonstrated increased 5LO expression and 5-hydroxyeicosatetraenoic acid (5HETE) and LTB4 secretion in their culture supernatant (Benard et al., 2014). Placentae from fetuses with congenital HCMV infection and brain damage showed expression of HCMV-immediate-early-antigen and 5LO when tested by immunohistochemistry (Benard et al., 2014). These findings suggest the lipoxygenase pathway’s role in the pathogenesis of congenital HCMV disease (Benard et al., 2014).

EBV interacts with monocytes to actively enhance the activation of the 5LO and the release of the formation of LTB4 and LTC4 (Gosselin and Borgeat, 1997). This activation was dependent on the viral binding as the effect of EBV was abolished by prior treatment of viral particles by heat or by an antibody raised against the glycoprotein gp350 of the viral envelope, but not by UV irradiation of the viral particles (Gosselin and Borgeat, 1997). Exposure of mononuclear cells to EBV was also dependent upon cell stimulation with a second agonist that activates cytosolic phospholipase A2, the enzyme required to form essential lipid mediators of inflammation (Gosselin and Borgeat, 1997).

EBV infection increases the formation of pro-inflammatory leukotrienes in human peripheral mononuclear cells (Belfiore et al., 2007) and triggers the malignant transformation of lymphocytes to Burkitt’s lymphoma cells. These Burkitt’s lymphoma cells are characterized by increased resistance to apoptosis (Gosselin and Borgeat, 1997). These cells overexpressed 5LO and 15LO, and studies with inhibitors show that 5LO and other LO-isoforms might be involved in EBV-mediated lymphoma progression. LTB4 has been can activate innate immunity and decrease the proliferation of EBV-induced induced B cells (Klein et al., 2008). LTB4 and its receptor BLT1 mediated LTB4-BLT1 lipid chemoattractant pathway can induce T-cell activation by enrichment of activation markers CD38 and HLA-DR and also express effector cytokines (IFN-γ, IL4) and inflammatory chemokine receptors (CCR1, CCR2, CCR6, and CXCR1) and subsequently inhibit the EBV-induced proliferation of B lymphocytes (Lin et al., 2008) is vital in early effector T-cell recruitment in mouse models of inflammation. BLT1 (+) T cells are enriched for (Chu et al., 2008).

EBV has been shown to bind to human neutrophils and stimulate homotypic aggregation, total RNA synthesis, and expression of the chemokines IL8 and macrophage inflammatory protein 1α (MIP1α) (Roberge et al., 1998). Neutrophils get primed with granulocyte-macrophage colony-stimulating factor (GMCSF), and treatment of neutrophils with GMCSF before EBV activation enhanced the production of LTB4 along with both chemokines MIP1α and IL8 (Roberge et al., 1998). EBV infection-induced neutrophils mediated induction of chemotactic cytokines, and LTB4 may improve its ability to infect B and T lymphocytes *via* increased recruitment to infection (Roberge et al., 1998).

EBV-transformed human B cells have been demonstrated to alter membrane phospholipid metabolism and intracellular calcium levels to modulate another potent mediator of the inflammatory response called platelet-activating factor (PAF) (Schulam et al., 1991). PAF receptors are expressed on Burkitt and non-Burkitt B cell lymphoma cell lines (Travers et al., 1991). PAF binds to B cells and induced arachidonic acid release and 5-hydroxyeicosatetraenoic (HETE) acid production (Schulam et al., 1991). It is interesting to note that apoptosis-resistant EBV-converted Burkitt’s lymphoma clones overexpress 5- and 12- lipoxygenases. The resistance to apoptosis increased concurrently with 5LO expression. 5LO inhibition reduced peroxide level indicating that 5LO promotes oxidative stress in EBV+ cells, drives tumorigenesis in multiple cell types, and engages in lymphomagenesis (Belfiore et al., 2007).

Blockade the 5LO enzyme and LTB4 secretion significantly downregulated KSHV latent ORF73, immunomodulatory K5, viral macrophage inflammatory protein 1 (MIP-1), and viral MIP-2 gene expression (Sharma-Walia et al., 2014). Inhibiting 5LO had no consequence on the KSHV lytic genes such as ORF50, immediate early lytic K8, and viral interferon-regulatory factor 2 (Sharma-Walia et al., 2014). Surprisingly, restraining 5LO activation diminished TH2 but elevated TH1-related cytokine secretion (Sharma-Walia et al., 2014). Blocking 5LO abrogated human monocyte recruitment, adhesion, and transendothelial migration of KSHV infected cells (Sharma-Walia et al., 2014). 5LO inhibition decreased fatty acid synthase (FASN) promoter activity and gene expression, much needed in lipogenesis during KSHV latency (Sharma-Walia et al., 2014).

### Boosting Membrane Lipid Anti-inflammatory Pathways

Pieces of evidence from recent studies indicate that lipid mediators play an essential role in pro-inflammation and inflammation resolution. Among these are resolvins, lipoxins, aspirin-triggered lipoxins (ATLs), which are increasingly being used to treat diseases such as metabolic diseases, diabetes, cardiovascular diseases, cancers herpesviruses. Lipoxins (LXs), an endogenously produced lipoxygenase interaction products of arachidonic acid metabolism, actively restore homeostasis by signaling metabolic and cellular action.

Lipoxins gets synthesized transcellular, in a bidirectional enzyme-mediated process. Arachidonic acid (AA) is converted to 15-HEPTE using the coordinated activity of 5LO in neutrophils and a closely allied enzyme, which is either 12LO or 15LO from platelet and endothelial cell, respectively. 15HEPTE, when acted upon by 5LO/12LO, synthesizes either LXA4 or LXB4 (Serhan et al., 1984). Additionally, lipoxin epimers, including aspirin-triggered lipoxin (ATL) are formed under the influence of aspirin treatment, as described by Serhan et al. (Chiang et al., 2004).

Aspirin has been the oldest of all successful non-steroidal anti-inflammatory drug (NSAID), analgesic-antipyretic therapeutic available for human usage. Aspirin acetylates serine residue on the active site of COX1 and COX2 in an irreversible manner. Aspirin mediated inhibition of COX2 halts the formation of pro-inflammatory prostaglandins and helps create 15R-hydroxy eicosatetraenoic acid from AA. 15-(R)-HETE is metabolized by 5LO to endogenous novel carbon 15-epimers of lipoxins, called ATLs (Chiang et al., 1998). Epilipoxins are more stable stereoisomers of lipoxins, and its analogs could help develop drugs that would not possess an adverse effect associated with COX1 inhibition. Owing to their low IC50 value and high potency, lipoxins and epilipoxins serve as a safe alternative to Aspirin, which possesses side effects like ulcer formation, bronchoconstriction, and nephropathy (Isakson et al., 1995).

Studies done on human KS and PEL cells have proved that KSHV strategically promotes its latency and malignant transforming ability by suppressing the production of anti-inflammatory signaling agents such as LXA4. At the same time, KSHV fosters the production of pro-inflammatory cytokines, lipoxygenases, cyclooxygenase, and metabolites of the latter two classes of enzymes to increase further the infectivity of the virus (Schultz et al., 2002; Rosenfeld et al., 2006). Interestingly, the anti-inflammatory lipoxin LXA4 is downregulated during KSHV infection to facilitate infected cell survival. The use of AA pathway inhibitors or supplementing anti-inflammatory lipid mediators has been proposed as an effective alternative therapeutic (Chandrasekharan and Sharma-Walia, 2019). Treatment of the KSHV infected cells with LXA4 or 15-epi-LXA4 reverses this pro-malignancy profile of pro-inflammatory signaling by an ALX/FPR receptor (**Figure 2**) dependent mechanism (Bentz et al., 2015). Lipoxin also interacts with signaling molecules and transcription factors such as NF-κB, AP-1 consisting of a heterodimer between c-Fos and c-Jun, nerve growth factor-regulated factor (NGF) 1A binding protein 1, and PPAR γ (Wang et al., 2020).

At higher concentrations (>30 nmol/liter), Lipoxins have been shown to interact with the Aryl hydrocarbon receptor (AhR); after that, AhR enters the nucleus where it joins the AhR nuclear translocator (ARNT). AHR/ARNT complex binds to xenobiotic response elements to initiate transcription of genes, including SOCS2 (suppressor of cytokine signaling), and thus participates in immunomodulation (Flavell and Murray, 2000; Chu et al., 2008). These findings suggest that the lipoxins or their analogs must be tested in animal models to ascertain if they can be used to treat KSHV associated cancers such as KS and PEL. In a recent study (Cai et al., 2013), it has been postulated that KSHV infected endothelial cells, when treated with a high concentration of LXA4, led to the nuclear translocation of the AhR protein. In the same study, PEL nuclear lysates, when passed through the LXA4 affinity column, yielded elutes rich in cellular nucleosome complex proteins, including MDMs and SWI/SNF protein, as identified by LC/LC-MS. Further, in the study, it was suggested that the nuclear translocation of AhR was responsible for the affinity interaction of cellular nucleosome complex proteins to LXA4. These nucleosome complex proteins play a significant role in chromatin modulation and the lytic induction of KSHV (Cai et al., 2013). These findings are suggestive of the prospect of lipoxins to be used for lytic induction therapy.

Mass spectrometry analysis from our experiment also suggested LXA4 be interacting with factors associated with metabolic and signaling pathways, immune response, cell proliferation, angiogenesis, transport-related proteins, and nuclear protein related to KSHV replication and life cycle (Asha et al., 2020). In the study, we identified that during the KSHV infection, RTA (replication and transcription activator) protein regulating the latent-lytic switch, recruits SWI/SNF chromatin remodeling complex and other cofactors to the viral promoter to facilitate RTA dependent KSHV gene expression. We also observed overexpression of SMARCB1 in KSHV infected skin and cells. SMARCB1 is expected to be associated with the lytic phase of KSHV. Studies have also proved that inhibitors related to these signaling pathways (mTOR) help control KSHV production (Feng et al., 2004). Keeping all these findings in mind, we speculate lipoxin as a very potent therapeutic agent against KSHV.

Among lipid mediators, many resolvins such as RvD1, epimer aspirin-triggered RvD1 (AT-RvD1), RvE1, neuroprotectin D1, and 11(12)-EET have been reported to serve as a novel antiviral drug for HSV-1 infections (Rajasagi et al., 2017; Zhang et al., 2020). Latency switch from the latent to the lytic cycle not only kills tumor cells but also triggers the immune response in lymphoma associated with EBV. NF-κB, a transcription factor and critical player of inflammation, is often activated in EBV infection. Previous studies have shown that aspirin treatment of EBV-positive lymphoma decreases nuclear translocation of NF-κB and promotes the lytic cycle. Aspirin, along with other anticancer drugs, could effectively treat EBV-positive lymphomas (Li et al., 2010).

## Conclusion and Future Perspective

Usage of new therapeutic strategies against herpesvirus infection still needs to meet challenges concerning their specificity, broad-spectrum effects, dosage requirement, toxicity, and in clinical trials. Co-infections with multiple viruses, parasites, malarial vector mosquitoes, and periodontal pathogens and antiviral resistance and the emergence of resistant viruses add to the complexity and severity of disease pathogenesis in immunocompromised patients, children, and neonates. Therefore, the development of newer and safer agents with novel targets with improved potency, lesser off-target, and side-effects is needed. Monotherapy fails in the immunocompromised patients as prolonged therapies are associated with the risk of antiviral resistance and combination antiviral therapy is preferred and more efficacious choice. Proinflammatory pathways regulate multiple aspects of inflammation, production of cytokines/chemokines/interferons, various immune cells [macrophages, dendritic cells, T cells, cytotoxic T lymphocytes (CTLs), Tregs, Th1/Th2 immunity, myeloid-derived suppressor cells], immunometabolism, and immune cell recruitment. COX2/PGE2 axis inhibition using COX inhibitors or PGE2 receptors antagonists along with PD-1 blockade has emerged as a promising adjunct therapy that had additive effects in enhancing CTL function, numbers, could rescue CTL exhaustion in cancer and warrants testing in herpesvirus related malignancies (Chen et al., 2015). LTB4 is often the first responder to infection, and LTB4/BLT1 has implications in immune cell recruitment and activation, chemokine skewing, CD8^+^ T cell recruitment initiating anti-tumor immunity, and blocking LTB4/BLT1 attenuates neutrophilic inflammation (Jala et al., 2017). Many LTB4 pathway drugs are in Phase 2 trials and offer innovative therapeutic opportunities for viral infections and their associated malignancies.

The unresolved chronic inflammation associated with herpesvirus infection leads to the production of reactive oxygen species (ROS), hypoxia, extracellular acidosis, and remodels microenvironment and reprograms the metabolism and recruitment of immune cells abrogating the efficacy of anticancer drugs and this might be the same scenario during infection (**Figure 1**). Recent advancement in understanding of the resolution-based pharmacology to resolve chronic inflammation has underscored the importance of safer anti-inflammatory drugs (lipoxins, aspirin-triggered lipoxins, resolvins, and their synthetic analogs) with versatile resolution properties. These drugs are immunomodulatory as their receptors are present on immune cells (innate lymphoid, NK, T, and B cells). These anti-inflammatory drugs can control CD4^+^ T cell differentiation into Th1 and Th17 effectors, decrease production of IL-2, IFN-γ, and TNF-α by CD8^+^ T cells. Since there is no perfect drug or magic bullet for herpesviral infections, we speculate that anti-inflammatory drugs from lipid targeting pathways would be beneficial as adjuvants to other antivirals, conventional and immune-based therapies (Zhang et al., 2017). Their targetable nuclear (NR4A2, ALXR/FPR, AhR) and plasma membrane (EP1-4, BLT1, BLT2) receptors (**Figure 2B**) may provide a breakthrough in herpesviral infection interventions (Zhang et al., 2017).

## Author Contributions

All authors contributed to the article and approved the submitted version. NS-W apologizes to all the colleagues whose work could not be cited in this manuscript.

## Funding

We are grateful for funding from NIH-funded grant R01CA 192970 to NS-W. The funders had no role in the design, decision to publish, or preparation of the manuscript.

## Conflict of Interest

The authors declare that the research was conducted in the absence of any commercial or financial relationships that could be construed as a potential conflict of interest.

## Abbreviations

AhR, Aryl hydrocarbon receptor; ARNT, AhR nuclear translocator; COX, cyclooxygenase; CCR, chemokine receptors; ChIP, chromatin immune-precipitation; CTL, cytotoxic T lymphocytes; EBV, Epstein-Barr virus; EZH2, enhancer of zeste homolog 2; GMCSF, granulocyte-macrophage colony-stimulating factor; HCMV, human cytomegalovirus; HDM, Histone demethylase; HMT, histone methyltransferase; HSV, herpes simplex virus; ICAM-1, intercellular adhesion molecule 1; KAP1, KRAB-associated protein 1; KICS, KSHV inflammatory cytokines syndrome; KSHV, Kaposi’s sarcoma herpesvirus; LO, lipoxygenase; mTOR, mammalian target of rapamycin; NGS, next-generation sequencing; NSAIDs, nonsteroidal anti-inflammatory drugs; PD-1, programmed cell death protein-1; PDL-1, programmed cell death ligand-1; PEL, primary effusion lymphoma PG, prostaglandin; PRC2, polycomb repressive complex 2; RTA, replication and transcription activator; SOCS, suppressor of cytokine signaling; TME, Tumor microenvironment; VZV, Varicella Zoster virus.
